# Metabolomics Analysis Reveals Novel Targets of Chemosensitizing Polyphenols and Omega-3 Polyunsaturated Fatty Acids in Triple Negative Breast Cancer Cells

**DOI:** 10.3390/ijms24054406

**Published:** 2023-02-23

**Authors:** Blake R. Rushing, Alleigh Wiggs, Sabrina Molina, Madison Schroder, Susan Sumner

**Affiliations:** 1 Nutrition Research Institute, University of North Carolina at Chapel Hill, Kannapolis, NC 28081, USA; 2 Department of Nutrition, University of North Carolina at Chapel Hill, Chapel Hill, NC 27599, USA

**Keywords:** triple negative breast cancer, metabolomics, drug response, polyphenols, omega-3 polyunsaturated fatty acids

## Abstract

Triple negative breast cancer (TNBC) is a subtype of breast cancer with typically poorer outcomes due to its aggressive clinical behavior and lack of targeted treatment options. Currently, treatment is limited to the administration of high-dose chemotherapeutics, which results in significant toxicities and drug resistance. As such, there is a need to de-escalate chemotherapeutic doses in TNBC while also retaining/improving treatment efficacy. Dietary polyphenols and omega-3 polyunsaturated fatty acids (PUFAs) have been demonstrated to have unique properties in experimental models of TNBC, improving the efficacy of doxorubicin and reversing multi-drug resistance. However, the pleiotropic nature of these compounds has caused their mechanisms to remain elusive, preventing the development of more potent mimetics to take advantage of their properties. Using untargeted metabolomics, we identify a diverse set of metabolites/metabolic pathways that are targeted by these compounds following treatment in MDA-MB-231 cells. Furthermore, we demonstrate that these chemosensitizers do not all target the same metabolic processes, but rather organize into distinct clusters based on similarities among metabolic targets. Common themes in metabolic targets included amino acid metabolism (particularly one-carbon and glutamine metabolism) and alterations in fatty acid oxidation. Moreover, doxorubicin treatment alone generally targeted different metabolites/pathways than chemosensitizers. This information provides novel insights into chemosensitization mechanisms in TNBC.

## 1. Introduction

Breast cancer is the most commonly diagnosed cancer in women and is the second leading cause of cancer-related deaths in women [1]. Triple negative breast cancer (TNBC) is a notoriously aggressive and highly metastatic classification of breast cancer characterized by a lack of expression of estrogen receptor (ER), progesterone receptor (PR), and human epidermal growth factor receptor 2 (HER2). TNBC accounts for approximately 15–20% of all breast cancer cases and has lower survival rates compared to hormone receptor-positive breast cancers due to a greater risk of recurrence and a more aggressive disease course [2,3]. Because TNBC does not respond to hormone therapy or HER2 directed therapeutics, treatment is limited to an aggressive course of cytotoxic chemotherapeutic drugs, which commonly includes high doses of anthracycline and taxane-based regimens [3]. This therapeutic strategy has significant health impacts, including issues with future fertility, premature menopause, cardiovascular toxicity, cognitive dysfunction, and poorer bone health [4]. As opposed to ER/PR-positive and HER2-positive breast cancers, few new treatment options have emerged for TNBC, resulting in little improvement in overall survival rates over the past 20–30 years compared to other cancers [5]. Recently, a panel of experts came up with a list of the top research needs in breast cancer, which was published in Annals of Oncology. This list included (1) improvement of care in young patients with breast cancer (due to higher rates of TNBC), (2) identification/validation of targets mediating chemotherapy resistance, and (3) identification of new targets in TNBC [6]. This highlights the necessity to explore new treatment options and/or methods to de-escalate chemotherapy in TNBC.

Certain dietary factors, such as polyphenols and omega-3 polyunsaturated fatty acids (PUFAs), have been shown to enhance the efficacy of chemotherapeutics or reverse multi-drug resistance (MDR) in vitro and in vivo against various cancers [7,8,9]. In particular, tannic acid [10], resveratrol [11,12], genistein [13], quercetin [14,15], curcumin [16], docosahexaenoic acid (DHA) [17], and eicosapentaenoic acid (EPA) [17] have been shown to increase the efficacy of doxorubicin—a first-line drug for TNBC—in MDA-MB-231 and MDA-MB-468 TNBC cell lines [10,11,12,13,14,15,16,18,19,20]. Moreover, these compounds have been shown to reverse MDR in cell lines of TNBC or other cancers [21,22,23,24,25,26,27,28]. A limitation of these compounds, particularly polyphenols, is their low bioavailability due to poor absorption or metabolic degradation by host/microbial enzymes [7,29,30], although major advancements have been made to overcome these issues using delivery systems such as nanoparticles or liposomes [31,32]. Nonetheless, these compounds still provide an excellent model to uncover novel therapeutic targets to enhance chemotherapeutic efficacy and/or reverse MDR. These dietary compounds are well-tolerated in humans and, therefore, therapeutics mimicking their actions (with improved pharmacokinetic properties) are likely to have favorable toxicity profiles. However, the mechanisms by which these compounds chemosensitize TNBC and reverse MDR remain unclear due to their pleiotropic nature. Indeed, many targets have been identified for these compounds, and it is ambiguous which targets are most crucial for their therapeutic effects [33,34,35,36].

**Figure 1:**
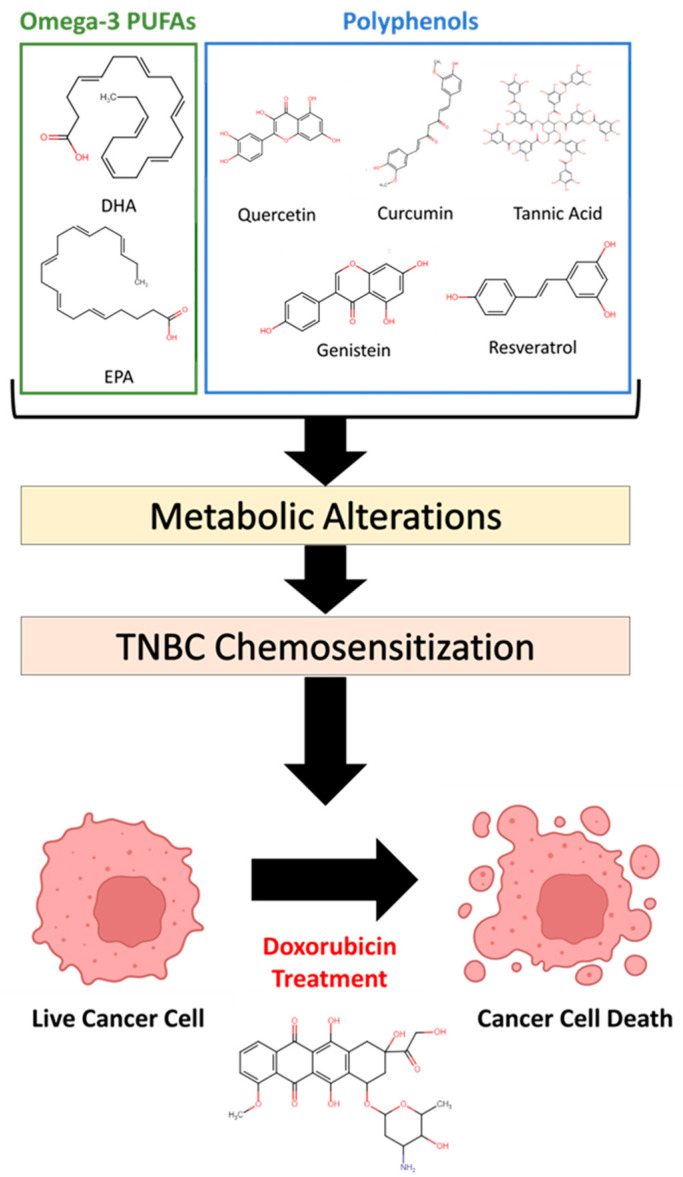
Chemical structures of chemosensitizing nutrients/dietary compounds used in this study. The goal of the current investigation is to use metabolomics to determine the metabolic targets of this panel of compounds to uncover potential mechanisms by which these substances sensitize triple negative breast cancer (TNBC) cells to chemotherapeutics.

Polyphenols and omega-3 PUFAs are highly studied molecules found in the diet, and much research has investigated their effects on cancer cells, including TNBC cells. In particular, these compounds and other anticancer nutrients/nutraceuticals have been shown to affect metabolic activity or processes regulating metabolism, which is associated with their anticancer activity [29,33,37,38]. Cancer cell metabolism greatly influences the response of cancer cells to therapeutics and the development of resistance, likely making metabolism a key target of these chemosensiziting compounds [39,40,41]. Moreover, co-administration of metabolic inhibitors has been shown to enhance the efficacy of chemotherapeutics [41], further strengthening the rationale that the metabolic targets of polyphenols/omega-3 PUFAs are critical for their chemosensitizing/MDR reversal effects. Research over many years has made it clear that these compounds affect multiple cellular targets, which has made it extremely difficult to identify the exact mechanisms of these nutrients using targeted methods. As a result, the literature is filled with various proposed mechanisms without a clear consensus on the critical targets of these compounds. Additionally, it is unknown whether the chemosensitizing effects of these compounds are due to targeting the same metabolic pathways as, or different ones from, TNBC chemotherapeutics (e.g., doxorubicin). Due to their pleiotropic properties, these nutrients/dietary compounds are well-positioned to be studied using omics techniques that can simultaneously measure many molecular species in a biological sample. In the current investigation, we present the use of metabolomics to elucidate metabolites/metabolic pathways targeted by a panel of polyphenols and PUFAs in the MDA-MB-231 TNBC cell line, providing insight towards the molecular mechanisms by which these compounds exert their chemosensitizing/MDR reversal effects (Figure 1).

## 2. Results

**Figure 2:**
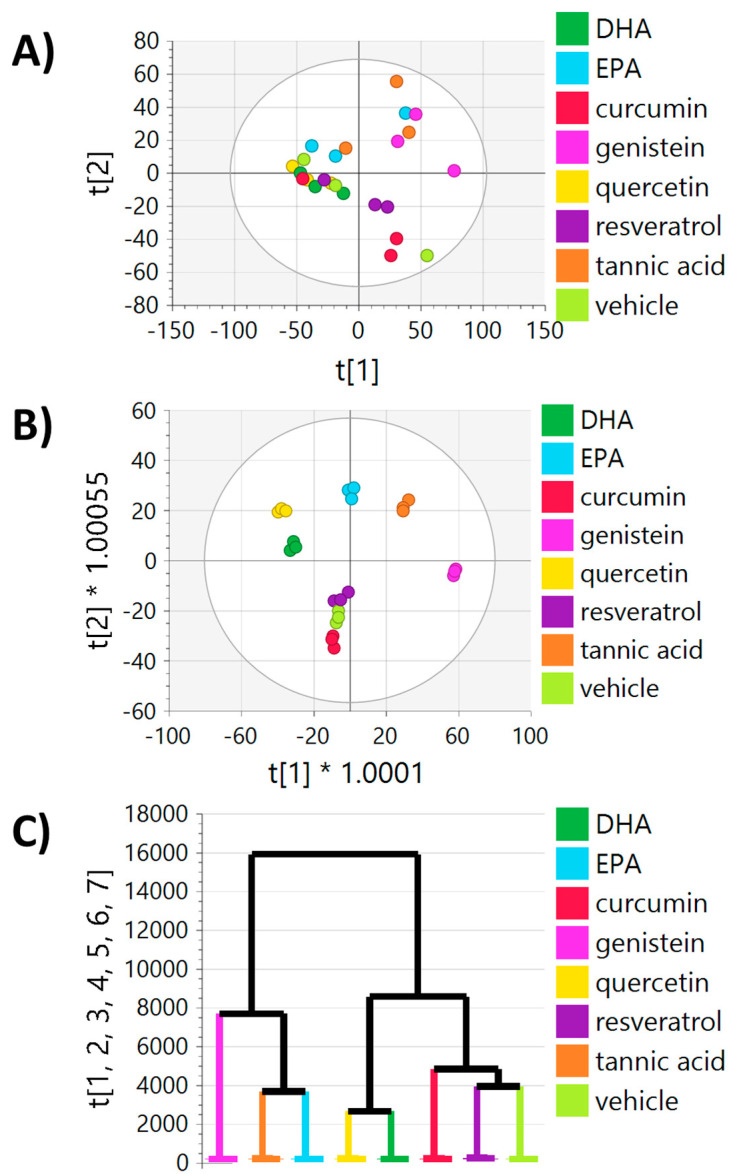
Multivariate analysis of all metabolomics peaks following 24 h of exposure of MDA-MB-231 cells to each treatment. (**A**) PCA and (B) OPLS-DA plots of cells treated with each chemosensitizer or vehicle control (DMSO). (**C**) Hierarchical clustering analysis of (**B**) showing treatments that give similar metabolic profiles. The X-axis represents the samples (colored by treatment) and the y-axis shows the similarity index calculated using all seven principal components of the OPLS-DA model. Distances between clusters calculated using the Ward method.

Following data preprocessing and filtering, 5097 peaks remained in the normalized metabolomics dataset, which were used for multivariate analyses. Quality control study pools (QCSPs) clustered tightly in the middle of the study samples, indicating the data collected was of sufficient quality (Figure S1). Principal component analysis (PCA) of all peaks showed moderate separation due to chemosensitizer treatment in MDA-MB-231 cells, with the largest separations seen with tannic acid, genistein, and EPA treatment compared to vehicle (Figure 2A). Orthogonal partial least squares-discriminant analysis (OPLS-DA), a supervised multivariate technique that uses class information, was able to produce very clear separation between treatment groups (Figure 2B). Importantly, this analysis showed treatments with similar metabolic profiles. For example, quercetin was closer in multivariate space to DHA compared to genistein, indicating that quercetin has a more similar metabotype with DHA. To further analyze overall similarities/differences in metabotypes between treatments, hierarchical clustering analysis (HCA) was performed on the OPLS-DA model to identify how treatments organized into clusters, identifying which treatments produced similar metabolic perturbations (Figure 2C). This clustering analysis revealed three distinct clusters: one cluster with resveratrol and curcumin, another cluster with DHA and quercetin, and a third cluster with tannic acid, EPA, and genistein. Notably, pairwise OPLS-DA comparisons between vehicle and each treatment group showed good model statistics with R2X, R2Y, and Q2 > 0.5, indicating that each treatment produced a robust effect on the metabolome of MDA-MB-231 cells, including treatment with doxorubicin (Supplementary Materials Table S1). These pairwise OPLS-DA models were used to calculate Variable Importance to Projection (VIP) scores for each peak, a multivariate score that indicates the contribution of a peak to the model. A full list of VIP scores, along with *p*-values and fold changes, for each vehicle–treatment combination is listed in Supplementary Materials Table S2. Furthermore, PCA of samples and QCSP replicates showed sufficient clustering and centering of QCSP samples, indicating good data quality.

**Figure 3:**
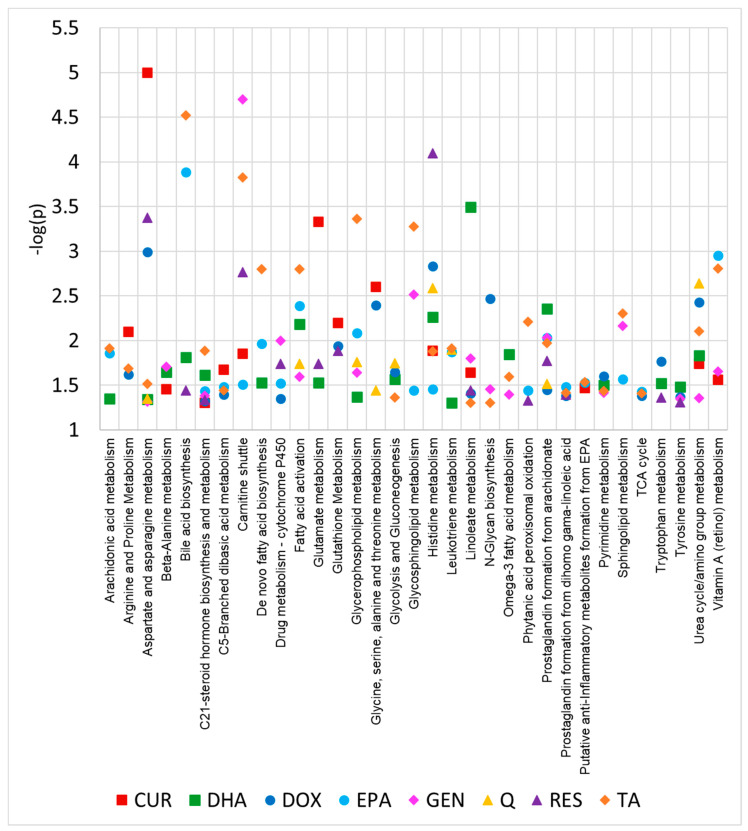
Significant pathways (*p* < 0.05) between vehicle and each treatment using all metabolomics peaks. Only pathways that are significant in three or more treatments are displayed (full list of significant pathways can be found in Table 1). Pathway analysis was conducted using MetaboAnalyst 5.0. CUR, curcumin; DOX, doxorubicin; GEN, genistein; Q, quercetin; RES, resveratrol; TA, tannic acid.

To better understand the metabolic targets of each treatment compound, pairwise pathway analyses were performed for each vehicle–treatment combination. For each comparison, all peaks with their corresponding *p*-values and fold changes were input into MetaboAnalyst 5.0 for pathway analysis. Significant pathways for each treatment are listed in Table 1. This analysis showed that several pathways were found to be altered by multiple treatments. Notably, C21-steroid hormone biosynthesis and metabolism, histidine metabolism, aspartate and asparagine metabolism, linoleate metabolism, prostaglandin formation from arachidonate, and urea cycle/amino group metabolism were found to be perturbed by five out of the eight treatments. A graphical representation of these results can be found in Figure 3, which plots the −log (*p*-value) for each pathway for each treatment (only pathways significant in three or more treatments are displayed). This analysis highlights that for most treatments, there are one or two pathways that are noticeably more affected than the rest. For example, curcumin seems to primarily affect aspartate and asparagine metabolism, genistein primarily affects the carnitine shuttle, and resveratrol primarily affects histidine metabolism; however, each treatment has significant activity in many other pathways, which is in agreement with the pleiotropic properties that have been reported for these compounds.

**Figure 4:**
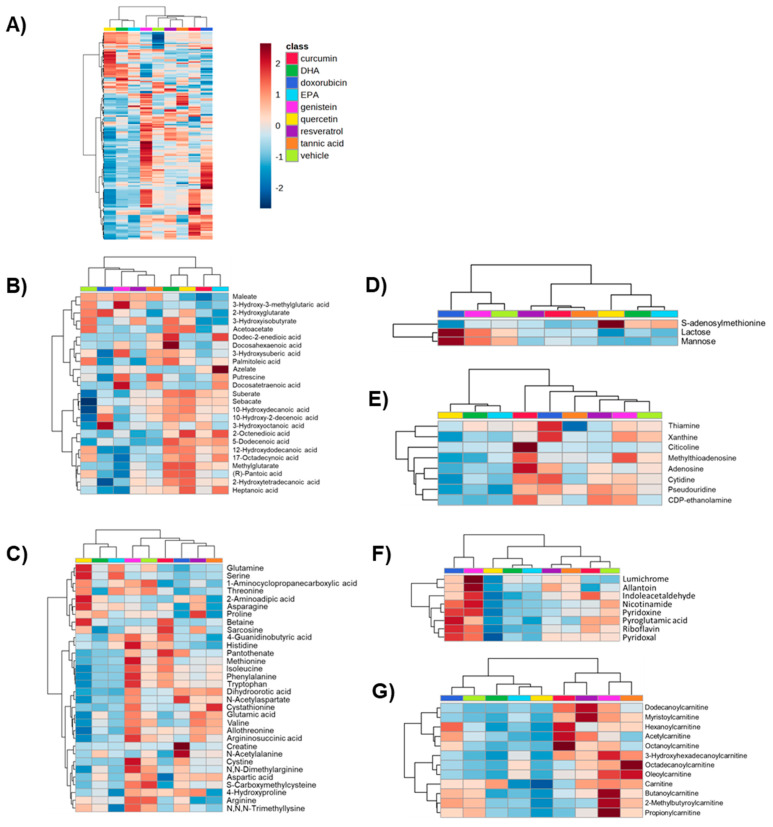
(**A**) Heatmap of all in-house matched metabolites across each treatment generated by MetaboAnalyst 5.0. Heatmaps were further subdivided based on Refmet categories: (**B**) fatty acyls, (**C**) organic acids, (**D**) carbohydrates, (**E**) nucleic acids, (**F**) organoheterocyclics, and (**G**) acylcarnitines. Hierarchical clustering was performed on samples using Euclidean distance measures. Heatmaps are auto-scaled (mean-centered and divided by standard deviation) for each metabolite.

While informative, MetaboAnalyst’s pathway analysis assigns metabolites to peaks based on accurate mass (MS) matches, which may lead to erroneous assignments due to lack of retention time (RT) and MS/MS matching. Because of this, we matched peaks to an in-house library of chemical reference standards that were run under identical instrument conditions, providing matches with increased evidence. From this, 169 peaks were matched to the in-house library at a level of OL1 (MS, RT, and MS/MS match), OL2a (RT and MS match), or OL2b (MS and MS/MS match). Displayed in Figure 4A is a heatmap of the in-house matched metabolites showing differences in abundance profiles across the different treatments. Clustering based on these metabolites leads to the similar grouping shown in Figure 2C using all of the metabolomics peaks, although EPA was shown to cluster with quercetin and DHA rather than tannic acid. ANOVA analysis of all in-house matched metabolites across all treatments was performed to identify compounds driving the observed clustering. Supplementary Materials Table S3 provides the *p*-values for all in-house metabolites using this analysis, revealing 58 in-house matched metabolites with an ANOVA of *p* < 0.05. Notable significant metabolites identified from this analysis were creatine, glutamine, DHA, docosatetraenoic acid, sphinganine, spermine, and putrescine, which all had *p*-values < 1 × 10^−5^. Pathway analysis was performed on all 58 of these significant metabolites, which identified glutathione metabolism, aminoacyl-tRNA biosynthesis, and arginine and proline metabolism as major metabolic pathways driving the clustering of treatments (Supplementary Materials Table S4).

To gain a better understanding of each treatment on specific metabolite groups, metabolites were subdivided into categories based on Refmet classifications [42] and heatmaps were generated for each category, which included fatty acyls (Figure 4B), organic acids (Figure 4C), carbohydrates (Figure 4D), nucleic acids (Figure 4E), organoheterocyclics (Figure 4F), and acylcarnitines—a subgroup of fatty acyls (Figure 4G). Clustering of each treatment in these category heatmaps provide more insight into the metabolic targets of each chemosensitizer based on distance from the vehicle-treated MDA-MB-231 cells. EPA, DHA, curcumin, and quercetin had the largest effect on fatty acyls, generally increasing the long-chain forms and decreasing the short-chain forms (Figure 4B). Quercetin, DHA, resveratrol, and tannic acid had the largest effect on organic acids, particularly quercetin, which showed strong increases in glutamine, serine, asparagine, and betaine relative to the vehicle. Other amino acids were generally decreased with treatment by these four compounds; however, genistein, despite clustering closely with the vehicle, showed strong increases in some amino acids including histidine, methionine, isoleucine, phenylalanine, tryptophan, and cystine (Figure 4C). For carbohydrates, quercetin, DHA, and EPA had the largest effect, with increases in S-adenosylmethionine and decreases in mannose and lactose (Figure 4D). Quercetin, DHA, EPA, and curcumin had the largest effect on nucleic acids, with the former three decreasing and the latter increasing these metabolites (Figure 4E). For organoheterocyclics (a class that includes many B vitamin forms), quercetin and genistein had the largest effects, with the former leading to increases and the latter leading to decreases in these metabolites (Figure 4F). Finally, acylcarnitines were strongly increased in curcumin, resveratrol, genistein, and tannic acid, particularly medium- and long-chain acylcarnitines (Figure 4G).

**Figure 5:**
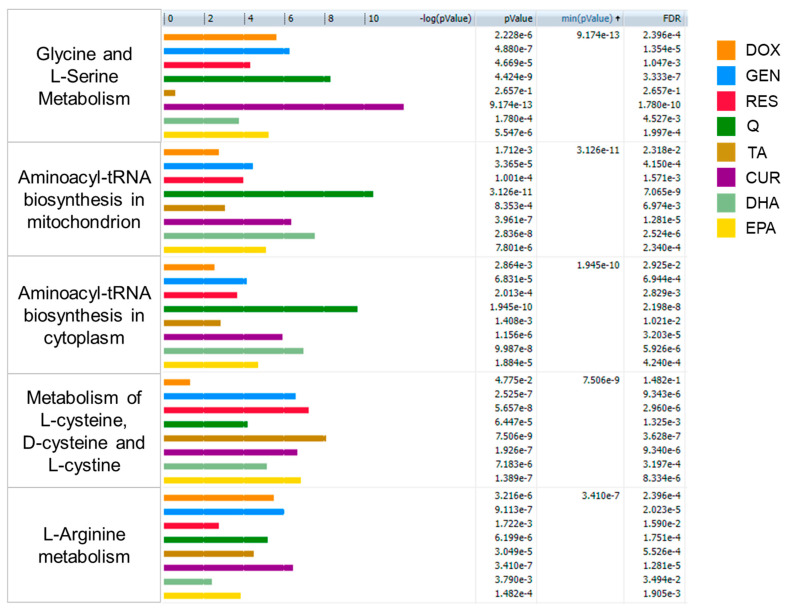
Top five most significant pathways calculated in GeneGo Metacore using all in-house matched metabolites. Pathway *p*-values (nominal and FDR corrected) are provided for each treatment. Pathways are ordered based on minimum *p*-value—the lowest *p*-value achieved by any of the treatments.

To provide more robust pathway analysis, in-house matched metabolites with fold changes for each treatment were input into GeneGo Metacore for pathway analysis. Metabolites with a VIP > 1 for a given vehicle–treatment comparison were considered significant and used for pathway mapping. Figure 5 displays the top five metabolic pathways identified by this analysis (Supplementary Materials Table S5 contains the full list of pathways). This analysis identified several amino acid-related pathways as significantly altered by the treatments, including pathways related to glycine, serine, arginine, cysteine, glutathione, and aminoacyl tRNAs. This agrees with the MetaboAnalyst results in Figure 3, which also identified glycine, serine, arginine, and glutathione pathways as significantly altered by treatment. Notably, the GeneGo Metacore analysis identified fewer fatty acid/cholesterol-related pathways compared to the MetaboAnalyst results, which may be due to the GeneGo Metacore analysis only using the in-house matched metabolites, which had a higher representation of amino acids and their metabolites.

## 3. Discussion

**Figure 6:**
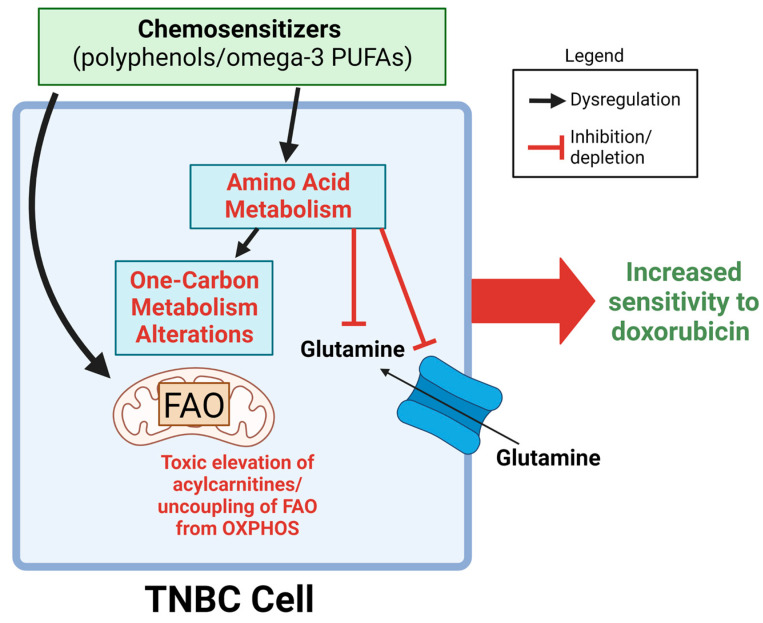
Proposed schema for chemosensitization of TNBC cells by polyphenols/omega-3 PUFAs. Metabolic targets of chemosensitizers were primarily focused towards amino acid metabolism and fatty acid oxidation. Chemosensitizers were observed to deplete glutamine levels, dysregulate amino acids in one-carbon metabolism, and (particularly for curcumin, resveratrol, tannic acid, and genistein) elevate acylcarnitines. These collective metabolic perturbations may underlie the chemosensitizing properties of these nutrients/dietary compounds. FAO; fatty acid oxidation; OXPHOS, oxidative phosphorylation. Figure created with BioRender.com.

Metabolic reprogramming is a hallmark of cancer and leads to cancer cells having distinct metabolic profiles compared to normal cells. This is due to cancer cells rewiring metabolic processes to overcome regulatory systems that would otherwise limit their growth and survival. This reprogramming also occurs in response to stressors, such as chemotherapy treatment, to promote the survival of cancer cells. In this way, alterations in cellular metabolism can modulate the response of cancer cells to drug treatment [43,44]. Certain nutrients/phytochemicals such as omega-3 PUFAs (often found in fish, nuts, and seeds) and polyphenols (commonly found in fruits, vegetables, nuts, and whole grains) have received significant interest in the research community for their observed health effects, such as their ability to prevent cancer and/or induce cancer cell death in experimental systems [34,45]. Included in these observations is the ability of these compounds to enhance the anticancer effect of chemotherapeutics. Because drug response is closely linked to cancer cell metabolism, we hypothesize that this chemosensitization effect is due to these compounds altering the metabotype of cancer cells, making them more responsive to the cytotoxic effect of chemotherapeutics. In the current investigation, we investigated a panel of polyphenols and omega-3 PUFAs that have previously been shown to increase the anticancer effect of doxorubicin in triple negative breast cancer cells. Using an untargeted metabolomics approach, we sought to identify the metabolites/metabolic pathways that are targeted by these chemosensitizing compounds (Figure 6).

Importantly, our findings indicated that the metabolic effects of these chemosensitizing compounds were broad, and often distinct from one another. This is in agreement with many studies that indicated that these compounds are pleiotropic. Additionally, this also suggests that there are multiple mechanisms by which metabolism can be altered to improve drug response. Even EPA and DHA, which are highly related metabolites that belong to the same metabolic pathway, showed different metabolic effects, although clustering analyses frequently placed these two treatments into the same cluster. This agrees with previous studies that have shown that EPA and DHA can have different anticancer effects, with DHA often shown to have greater anticancer effects than EPA [37]. Notably, doxorubicin-treated MDA-MB-231 cells generally showed very different metabolic profiles than chemosensitizer-treated cells. This suggests that these polyphenols and omega-3 PUFAs sensitize TNBC cells by targeting different, complementary metabolites to increase the drug’s cytotoxic effect.

Pathway analyses provide a means to more easily interpret overall biological effects in metabolomics data. Herein, we provided two pathway analyses: one using all peaks via MetaboAnalyst and another using only in-house matched metabolites via GeneGo. Both analyses indicated amino acid metabolism as a major target, with the GeneGo results (using only matches with the highest evidence basis) particularly identifying amino acids involved in one-carbon metabolism (glycine, serine, cysteine, and cystine)—a pathway that controls the flux of one-carbon units towards numerous pathways including nucleotide and lipid metabolism [46]. Cancer cells are particularly sensitive to deprivation of one-carbon units through nutrient restriction or pharmacological inhibition of the one-carbon metabolic pathway, as seen with the clinical success of folate inhibitors such as methotrexate and pemetrexed [47]. Indeed, cancer cells rely on this pathway to increase anabolic pathways (nucleotide/lipid synthesis), produce NADPH to adapt to the high levels of reactive oxygen species that are characteristic of cancer cells, produce energy in the form of adenosine triphosphate (ATP), and alter DNA methylation patterns [46,47]. Interestingly, the direction of change of metabolites in this pathway varied across treatments, suggesting that dysregulation of this pathway—by either increasing or decreasing activity—can lead to chemosensitization towards doxorubicin treatment.

Another amino acid that was heavily affected by chemosensitizer treatment was glutamine, which was decreased in all chemosensitizer treatments. Conversely, glutamine levels were strongly increased following doxorubicin treatment (VIP > 1, *p* = 8.61 × 10^−5^, fold change > 5) (Supplementary Materials Table S2). This was one of the few instances where an OL1 metabolite was consistently changed in the same direction by all chemosensitizers while also being significantly affected by doxorubicin treatment. Increases in glutamine uptake are commonly seen in cancers to support biosynthetic reactions and combat redox stress, and yield these results by replenishing TCA cycle intermediates, which are then shuttled to anabolic reactions [48]. The observation that glutamine was increased following doxorubicin treatment may be an indicator that increased glutamine uptake is a stress response that TNBC cells undergo to survive the cytotoxic effects of this drug. Consequently, this panel of polyphenols/omega-3 PUFAs may enhance doxorubicin’s cytotoxic effect by depleting glutamine levels, preventing this survival response. In addition to glutamine, three other in-house matched metabolites were altered in all treatment groups (VIP > 1): myristoylcarnitine, octadecanoylcarnitine, and 3-hydroxyhexadecanoylcarnitine (Supplementary Materials Table S2). This indicates that these acylcarnitines and glutamine are shared targets of doxorubicin and these chemosensitizers, and that the simultaneous disruption of these metabolic pathways during doxorubicin treatment may lead to increased drug efficacy. Future studies are needed to investigate the metabolic profiles of cells co-treated with doxorubicin and these chemosensitizers to determine if these effects are seen when both agents are administered simultaneously. In addition, while previous studies have shown an increase in cytotoxicity from these co-treatments in TNBC cells, we did not assess cell viability with these combinations, which should be confirmed in future studies. Future studies should also investigate if these metabolic processes are targeted by other polyphenols/PUFAs beyond those used in this study. Lastly, additional TNBC models should be studied to confirm our results, as the current investigation only assessed the effects of these chemosensitizers in the MDA-MB-231 cell line.

One of the observed effects of polyphenols on cancer cells is their ability to modulate cellular energetics. Polyphenols have been shown to activate AMPK, which alters many anabolic and catabolic processes, such as fatty acid oxidation, glycolysis, lipogenesis, and autophagy [49]. Cancer cells carefully balance energy consumption and generating pathways to sustain increased proliferation and manage reactive oxygen species (ROS) levels [50]. Disrupting this balance may be a mechanism by which polyphenols cause cancer cell death and/or increase chemotherapeutic efficacy. Our findings that a subset of polyphenols (curcumin, genistein, tannic acid, and resveratrol) greatly alters acylcarnitine levels in TNBC cells, typically increasing their levels. Acylcarnitines are intermediates in fatty acid oxidation that are formed from acyl-CoA and carnitine by the action of carnitine palmitoyltransferase 1 (CPT-1). Once formed, acylcarnitines are able to pass into the mitochondrial matrix, where they are re-converted into acyl-CoA by CPT-2 and then oxidized via β-oxidation to acetyl-CoA, which is then used in the TCA cycle for ATP production [51]. In the context of diabetes, disturbances in the acylcarnitine pool have been shown to be established markers of mitochondrial dysfunction and the uncoupling of fatty acid oxidation (FAO) from oxidative phosphorylation [52]. Indeed, elevation of acylcarnitines has been shown to occur when FAO activity outpaces the TCA cycle, leading to increased lipolysis and incomplete mitochondrial substrate oxidation [53,54]. Under these conditions, where substrate catabolism exceeds ATP demand, the increased reducing pressure of the cell (NADH, FADH_2_) on the electron transport chain leads to the generation of ROS (H_2_O_2_, •O_2_) [53]. Excessive mitochondrial ROS and accumulation of acyls in the mitochondria have been shown to open the mitochondrial permeability transition pore (PTP), causing cell death [52,55,56,57,58]. Our data suggest that a similar mechanism may occur in cancer cells following treatment with these polyphenols. Although polyphenols have historically been recognized as antioxidants, they are now recognized to have pro-oxidant effects in cancer cell environments [59,60,61,62], which may be due to the accumulation of acylcarnitines. Interestingly, the breast cancer cell response to doxorubicin is heavily influenced by mitochondrial activity, with factors such as mitochondrial oxidation state, depolarization, matrix calcium levels, and ROS production mediating its activity [63,64]. Although the exact mechanism of action of doxorubicin remains unclear, it has been well observed to lead to mitochondrial dysfunction, which is thought to play a critical role in the cardiotoxicity seen with this drug in in vitro, in vivo, and clinical studies [65]. Our observation that a subset of chemosensitizers heavily affects acylcarnitine levels suggests that these compounds increase doxorubicin efficacy in breast cancer cells by shifting the equilibrium of mitochondrial activity. In turn, this shifting of mitochondrial activity may lead to a cytoprotective effect of these compounds in cardiomyocytes against doxorubicin-mediated toxicity [66,67,68]. More research is needed to better understand if the metabolic effects of these compounds are also seen in cardiomyocytes and if they contribute to this observation of selective toxicity (cytotoxic in cancer cells, cytoprotective in healthy cells). The generally higher levels of ROS seen in cancer cells versus normal cells may play a role in determining this selective toxicity, making cancer cells more sensitive to imbalances in mitochondrial metabolism/ROS production [69]. Of note, acylcarnitine treatment has been shown to slow the development of certain cancers, such as colon cancer in vivo [70].

Additionally, polyphenols/omega-3 PUFAs have been shown to alter the activity of the PI3K-Akt/mTOR/AMPK signaling axis, which is well known to modulate amino acid metabolism and mitochondrial activity/fatty acid oxidation, providing a possible mechanism for how these chemosensitizing compounds affect the pathways seen in this study [37,71,72]. Indeed, the mTOR signaling pathway is a central metabolic regulator in the cell that senses the nutritional status of the cell, controlling growth and metabolic activity [73]. Previous studies have shown that modulation of mTOR activity in combination with doxorubicin shows synergistic activity in in vitro and in vivo models of various cancers [74,74,75,76]. This combination treatment has also been shown to be effective in a Phase I trial for the treatment of mesenchymal TNBC [77]. mTOR inhibition has also been shown to be effective in combination with other anticancer therapeutics, indicating that targeting of this pathway is a promising avenue for chemosensitization [78]. Because our panel of chemosensitizers shows different profiles of metabolic perturbations, it is possible that they target different locations on the mTOR pathway and/or have different off-target effects, which may contribute to their anticancer effects and/or favorable toxicity profiles.

Previous metabolic effects of these compounds in other disease contexts are in agreement with our results. Resveratrol, curcumin, genistein, and tannic acid have been shown to increase acylcarnitine profiles systemically and/or in skeletal muscle, alter the expression of mitochondrial β-oxidation enzymes, affect mitochondrial biogenesis, and change mitochondrial bioenergetics, which has been linked to the anti-obesogenic and anti-aging effects of these compounds [79,80,81,82,83,84,85]. Additionally, resveratrol, curcumin, genistein, quercetin, DHA, and EPA have all been shown to modulate glutaminolysis/glutamine levels in vitro or in vivo [86,87,88,89,90]. While these previous studies give additional validity to our results, this study was a screening approach to identify a list of metabolic targets of these compounds, and will need to be validated using targeted methods in additional model systems.

The pleiotropic effects of these polyphenols/omega-3 PUFAs highlight the promise of multi-targeted therapy in cancer. Indeed, the anticancer effect of these compounds may be attributed to a combination of effects on multiple metabolites/pathways rather than a single primary target. Our study uncovers novel metabolic targets of these compounds that aid in explaining their chemosensitizing effects, and these metabolic targets may provide an explanation for the health benefits of these compounds in other disease areas (aging, cardiovascular disease, neurodegeneration, etc). However, it should be noted that the concentrations, dose times, and cell model used in this study were specifically designed in the context of enhancing the effect of doxorubicin in TNBC, and therefore may not necessarily reflect the metabolic effects seen in these other contexts. Furthermore, our observation that each treatment generally produced unique metabolic profiles indicates that different polyphenols/omega-3 PUFAs target different sets of metabolites/metabolic pathways. This may provide a rationale for combining polyphenols/omega-3 PUFAs based on their targeting of similar/complementary metabolites to obtain an additive/synergistic effect—for example, resveratrol and genistein may be combined as a co-treatment since both increase acylcarnitines. Conversely, this may also be a way to predict compounds that could antagonize each other’s effects, leading to diminished therapeutic effects. Indeed, combinations of these compounds have been shown to be a promising area for improving their effects [7,30]; therefore, using omics technologies to make rationalized combinations is an area worthy of future study.

In conclusion, our study uncovered novel mechanisms by which polyphenols/omega-3 PUFAs target metabolism under doxorubicin-chemosensitizing conditions. While bioavailability issues continue to be a challenge in using these compounds clinically, mechanistic information, such as the data presented herein, may form a basis for developing mimetics with more favorable pharmacokinetic profiles. For TNBC specifically, understanding these chemosensitization mechanisms is clinically very valuable, as there is a great need to improve treatment outcomes and de-escalate drug doses to mitigate side effects. It is important to note that more information is needed concerning the mechanism of action of these polyphenols and omega-3 PUFAs, as well as more testing in experimental and clinical settings. These needs must be met to fully understand their anticancer properties and to use this information to improve outcomes for TNBC patients.

## 4. Materials and Methods

### 4.1. Chemical Reagents

Optima grade solvents (water with 0.1% formic acid and methanol with 0.1% formic acid) and fetal bovine serum (FBS) were purchased from Fisher Scientific (Waltham, MA, USA). Dubelcco’s Modified Eagle Medium (DMEM) with high glucose and phosphate buffered saline (PBS) was purchased from Gibco (Grand Island, NY, USA). Resveratrol, curcumin, quercetin, genistein, tannic acid, DHA, and EPA were purchased from Cayman Chemical (Ann Arbor, MI, USA). The MDA-MB-231 cell line was purchased from the American Type Culture Collection (ATCC) (Manassas, VA, USA).

### 4.2. Cell Culture

MDA-MB-231 cells were cultured according to manufacturer guidelines. Cells were cultured in DMEM supplemented with 10% FBS, 2 mM glutamine, 50 U/mL penicillin, and 50 µg/mL streptomycin. Cells were plated in 10 cm culture dishes and grown to ~70% confluency. Cells were treated with individual test compounds at concentrations previously shown to chemosensitize cells to doxorubicin (resveratrol—50 µM, curcumin—40 µM, tannic acid—25 µM, genistein—50 µM, DHA—29 µM, EPA—32µM) [10,11,12,13,14,15,16,17]. Concentration of the vehicle (dimethyl sulfoxide, DMSO) was kept at 0.01% for all treatments. Additional dishes were treated with vehicle alone and doxorubicin (0.2 µM). All treatments were performed in triplicate for 24 h.

### 4.3. Metabolite Extraction

After treatment, metabolites were extracted from cell samples as described previously [91,92,93]. Briefly, treatment media were aspirated, and cells were washed with 5 mL of ice-cold PBS. After aspirating off PBS, 2 mL of ice-cold 80% methanol was added to culture dishes, and cells were detached using cell scrapers. Cell suspensions were added to MagNA lyser homogenization tubes with ceramic beads inside and were lysed using an Omni Bead Ruptor Elite (OMNI International) at 6.00 m/s for two cycles at 45 s each with 30 s dwell time between each cycle. Additional 80% methanol was added to each tube to normalize for protein concentration. Samples were centrifuged at 16,000× *g* at 4 °C for 10 min and supernatants were transferred to autosampler vials for analysis by ultra-high-pressure liquid chromatography–high-resolution mass spectrometry (UHPLC-HRMS). Quality control study pools (QCSP) were created by combining 10 µL of each sample into a single mixture. Method blanks were created by adding 500 µL of 80% methanol to empty MagNA lyser tubes and were processed in an identical manner as the study samples.

### 4.4. UHPLC-HRMS Metabolomics Data Acquisition, Preprocessing, and Multivariate Analysis

Metabolomics data were acquired via previously published UHPLC-HRMS methods using a Vanquish UHPLC system coupled to a Q Exactive™ HF-X Hybrid Quadrupole-Orbitrap Mass Spectrometer (Thermo Fisher Scientific, San Jose, CA, USA) equipped with an HSS T3 C18 column (2.1 × 100 mm, 1.7 µm, Waters Corporation) held at 50 °C [91,92,93,94,95,96,97,98]. A binary pump was used with water + 0.1% formic acid (A) and methanol + 0.1% formic acid (B) as mobile phases. The mobile phase gradient started from 2% B, increased to 100% B in 16 min, and was then held for 4 min with a flow rate of 400 µL/min. Mass spectral data were collected using a data-dependent acquisition mode in positive polarity at 70–1050 m/z. QCSP and blank injections were placed at a rate of 10% throughout the study samples. An injection volume of 5 µL was used for analysis of each sample. Raw UHPLC-HRMS data were imported into Progenesis QI (version 2.1, Waters Corporation, MA, USA) for alignment, peak picking, and deconvolution. Background signals were removed by filtering out peaks with a higher average abundance in the blank injections as compared to the QCSP injections. Data were normalized using a QCSP reference sample using the “normalize to all” function in progenesis [99].

### 4.5. Multivariate and Univariate Statistical Analysis

The normalized, filtered data were imported into SIMCA 16 (Sartorius Stedim Data Analytics AB, Umeå, Sweden), scaled using Unit Variance (UV) scaling, and then used to generate principal component analysis (PCA) and orthogonal partial least squares-discriminant analysis (OPLS-DA). PCA plots were used to assess data quality and clustering of QCSP samples, and OPLS-DA plots were used to assess the separation of metabolomes between vehicle and treated cells as well as to calculate variable importance to projection (VIP) scores for each peak. Heatmaps were generated using MetaboAnalyst 5.0. Fold changes and *p*-values were calculated for each peak for each treatment as compared to the vehicle control. *p*-values were calculated using Student’s *t*-test. *p*-values were not adjusted for multiple testing due to the small sample size of this study and the exploratory, rather than confirmatory, nature of this study [100].

### 4.6. Compound Identification/Annotation

Peaks were matched to an in-house library of reference standards or public mass spectral databases from the National Institute of Standards and Technology (NIST) and METLIN. Peaks were matched to metabolites by retention time (RT, ±0.5 min, in-house library only), exact mass (MS, <5 ppm), and fragmentation pattern (MS/MS, similarity score > 30). An ontology system was given to denote the evidence basis for each metabolite assignment. OL1 refers to a match to the in-house library for RT, MS, and MS/MS; OL2a refers to an in-house match to the in-house library for RT and MS; OL2b refers to a match to the in-house library for MS and MS/MS; PDa refers to a match to public databases for MS and MS/MS; PDb refers to a public database match for MS and theoretical MS/MS (HMDB); PDc refers to a public database match for MS and isotopic similarity; PDd refers to a public database match for MS only.

### 4.7. Pathway Analysis

For each treatment–vehicle comparison, all untargeted peaks, along with their calculated *p*-values and fold changes, were imported into the “Functional Analysis” module in MetaboAnalyst 5.0 to identify significantly perturbed metabolic pathways. Both mummichog and gene set enrichment analysis (GSEA) algorithms were chosen for analysis. The default top 10% of peaks by *p*-value was chosen for the mummichog algorithm. For more robust pathway analysis, only metabolites that matched to levels of OL1, OL2a, and OL2b along with their fold changes were imported into GeneGo Metacore (Clarivate Analytics, London, UK), and pathway analysis was performed for metabolites that had a VIP > 1 in the pairwise OPLS-DA comparisons between vehicle and each treatment.

## Figures and Tables

**Table 1:** Significant pathways (*p* < 0.05) for each treatment calculated using MetaboAnalyst. Values reported are combined p-values calculated with MetaboAnalyst 5.0 using both the mummichog and GSEA pathway algorithms. The default top 10% of the p-values was selected for the mummichog algorithm.

	Doxorubicin	EPA	DHA	Quercetin	Genistein	Resveratrol	Tannic Acid	Curcumin
C21-steroid hormone biosynthesis and metabolism	4.7 × 10^−2^	3.6 × 10^−2^	2.4 × 10^−2^		4.1 × 10^−2^	4.6 × 10^−2^	1.3 × 10^−2^	4.9 × 10^−2^
Histidine metabolism	1.4 × 10^−3^	2.4 × 10^−2^	1.6 × 10^−2^	5.5 × 10^−3^		6.0 × 10^−5^	1.3 × 10^−2^	1.5 × 10^−2^
Aspartate and asparagine metabolism	1.0 × 10^−3^		4.5 × 10^−2^		4.8 × 10^−2^	5.3 × 10^−4^	3.0 × 10^−2^	<1.0 × 10^−5^
Linoleate metabolism	3.9 × 10^−2^		3.2 × 10^−4^		1.6 × 10^−2^	3.6 × 10^−2^	5.0 × 10^−2^	2.3 × 10^−2^
Prostaglandin formation from arachidonate	3.5 × 10^−2^	9.2 × 10^−3^	4.4 × 10^−3^		9.2 × 10^−3^	1.7 × 10^−2^	1.1 × 10^−2^	
Urea cycle/amino group metabolism	6.9 × 10^−3^		1.5 × 10^−2^	3.5 × 10^−2^	4.4 × 10^−2^		7.9 × 10^−3^	1.8 × 10^−2^
Carnitine shuttle		3.1 × 10^−2^			2.0 × 10^−5^	1.7 × 10^−3^	1.5 × 10^−4^	1.4 × 10^−2^
Drug metabolism—cytochrome P450	4.4 × 10^−2^	3.0 × 10^−2^			9.9 × 10^−3^	1.8 × 10^−2^	3.0 × 10^−2^	
Glycerophospholipid metabolism		8.2 × 10^−3^	4.2 × 10^−2^	4.5 × 10^−2^	2.3 × 10^−2^		4.3 × 10^−4^	
Pyrimidine metabolism	2.5 × 10^−2^	3.7 × 10^−2^	3.1 × 10^−2^		3.8 × 10^−2^		3.7 × 10^−2^	
Bile acid biosynthesis		1.3 × 10^−4^	1.5 × 10^−2^			2.3 × 10^−2^	3.0 × 10^−5^	
C5-Branched dibasic acid metabolism	3.4 × 10^−2^	3.2 × 10^−2^					3.5 × 10^−2^	2.1 × 10^−2^
Fatty acid activation		4.0 × 10^−3^	1.2 × 10^−2^		2.6 × 10^−2^		1.6 × 10^−3^	
Leukotriene metabolism		1.3 × 10^−2^	5.0 × 10^−2^	3.0 × 10^−2^			1.2 × 10^−2^	
N-Glycan biosynthesis	3.3 × 10^−3^	5.0 × 10^−2^			3.5 × 10^−2^		5.0 × 10^−2^	
Purine metabolism	3.3 × 10^−2^	4.6 × 10^−2^			3.3 × 10^−2^			4.3 × 10^−2^
Vitamin A (retinol) metabolism		1.1 × 10^−3^			2.2 × 10^−2^		1.6 × 10^−3^	2.8 × 10^−2^
Arachidonic acid metabolism		1.3 × 10^−2^	4.4 × 10^−2^				1.2 × 10^−2^	
De novo fatty acid biosynthesis		1.1 × 10^−2^	2.9 × 10^−2^				1.6 × 10^−3^	
Glutathione metabolism	1.6 × 10^−2^					8.7 × 10^−3^		9.0 × 10^−3^
Glycolysis and gluconeogenesis	2.2 × 10^−2^			4.7 × 10^−3^			4.3 × 10^−2^	
Glycosphingolipid metabolism		3.6 × 10^−2^			3.0 × 10^−3^		5.3 × 10^−4^	
Prostaglandin formation from dihomo gama-linoleic acid	3.9 × 10^−2^					4.0 × 10^−2^	3.6 × 10^−2^	
Putative anti-inflammatory metabolites formation from EPA		2.8 × 10^−2^					2.9 × 10^−2^	3.3 × 10^−2^
Sphingolipid metabolism		2.8 × 10^−2^			6.9 × 10^−3^		5.0 × 10^−3^	
Tryptophan metabolism	4.3 × 10^−2^		3.0 × 10^−2^			4.3 × 10^−2^		
Tyrosine metabolism	4.3 × 10^−2^		3.3 × 10^−2^		4.5 × 10^−2^			
Alanine and aspartate metabolism						4.7 × 10^−2^		6.5 × 10^−3^
Arginine and proline metabolism							2.0 × 10^−2^	7.7 × 10^−3^
Beta-Alanine metabolism					1.9 × 10^−2^			5.0 × 10^−2^
Fatty acid metabolism		9.6 × 10^−3^					8.6 × 10^−3^	
Fatty acid oxidation, peroxisome					2.4 × 10^−2^		9.4 × 10^−3^	
Glutamate metabolism						1.6 × 10^−2^		5.4 × 10^−4^
Glycine, serine, alanine and threonine metabolism	4.0 × 10^−3^							2.4 × 10^−3^
Methionine and cysteine metabolism	2.2 × 10^−2^		4.3 × 10^−2^					
Nitrogen metabolism				2.8 × 10^−2^		4.7 × 10^−2^		
Omega-3 fatty acid metabolism					2.9 × 10^−2^		2.6 × 10^−2^	
Pyruvate metabolism	4.6 × 10^−3^							5.0 × 10^−2^
Saturated fatty acid beta-oxidation					1.5 × 10^−2^		4.4 × 10^−2^	
Sialic acid metabolism		3.2 × 10^−2^			2.6 × 10^−2^			
Squalene and cholesterol biosynthesis		3.6 × 10^−2^					3.6 × 10^−2^	
TCA cycle		3.7 × 10^−2^					3.7 × 10^−2^	
Vitamin E metabolism		4.3 × 10^−2^					2.1 × 10^−2^	
3-oxo-10R-octadecatrienoate beta-oxidation								3.6 × 10^−2^
Aminosugars metabolism						4.3 × 10^−2^		
Blood group biosynthesis	2.3 × 10^−2^							
Carbon fixation				8.3 × 10^−3^				
Di-unsaturated fatty acid beta-oxidation			4.8 × 10^−2^					
Dimethyl-branched-chain fatty acid mitochondrial beta-oxidation						3.1 × 10^−2^		
Fructose and mannose metabolism	7.0 × 10^−3^							
Galactose metabolism	2.4 × 10^−2^							
Glycosphingolipid biosynthesis—ganglioseries							2.6 × 10^−2^	
Glycosphingolipid biosynthesis—lactoseries	2.3 × 10^−2^							
Glycosphingolipid biosynthesis—neolactoseries	2.3 × 10^−2^							
Hexose phosphorylation	1.7 × 10^−2^							
Limonene and pinene degradation						3.4 × 10^−2^		
Lysine metabolism	3.0 × 10^−2^							
Omega-6 fatty acid metabolism					2.1 × 10^−2^			
Pentose and glucuronate interconversions	4.9 × 10^−2^							
Pentose phosphate pathway	2.7 × 10^−2^							
Phytanic acid peroxisomal oxidation							1.1 × 10^−2^	
Polyunsaturated fatty acid biosynthesis					3.8 × 10^−2^			
Selenoamino acid metabolism			2.1 × 10^−2^					
Valine, leucine and isoleucine degradation						5.6 × 10^−3^		
Vitamin B3 (nicotinate and nicotinamide) metabolism	2.4 × 10^−2^							
Vitamin B9 (folate) metabolism			3.6 × 10^−2^					
Vitamin D3 (cholecalciferol) metabolism							3.6 × 10^−2^	
Vitamin K metabolism							2.1 × 10^−2^	

## Data Availability

All preprocessed data are available in the Supplementary Materials. Raw data are available upon request.

## Supplementary Materials

The supporting information can be downloaded at: https://www.mdpi.com/article/10.3390/ijms24054406/s1.
