# Supplementary material for: Metabolomics Analysis Reveals Novel Targets of Chemosensitizing Polyphenols and Omega-3 Polyunsaturated Fatty Acids in Triple Negative Breast Cancer Cells
Source: Int J Mol Sci. 2023 Feb 23;24(5):4406. doi: 10.3390/ijms24054406 (PMC10002396; doi:10.3390/ijms24054406)
Supplement: Supplementary file 1 [file ijms-24-04406-s001.zip › Supplemental Figure S1.pdf]

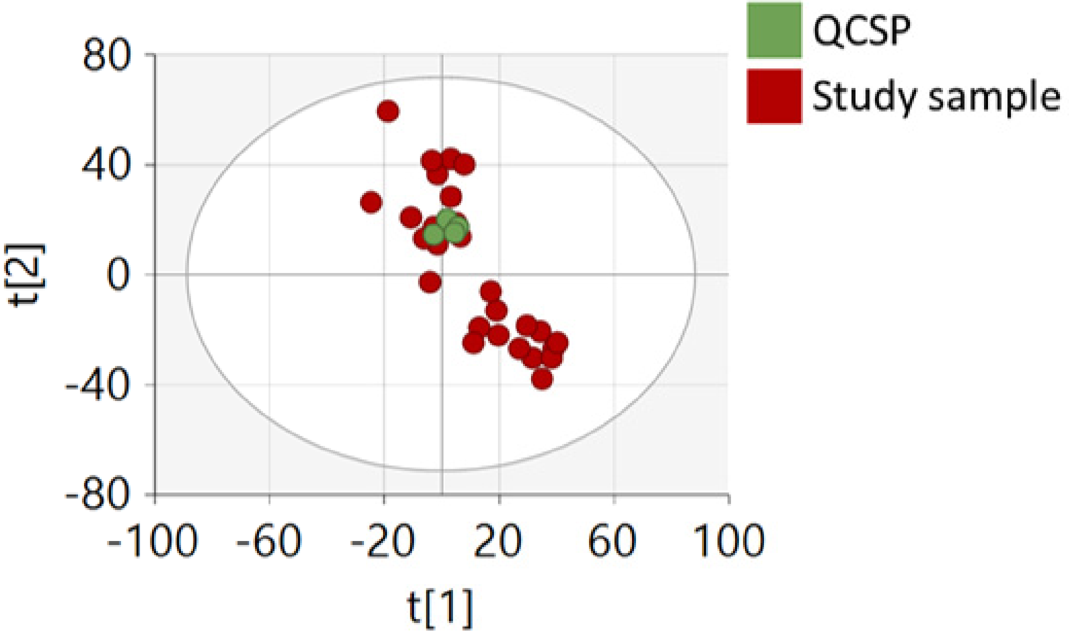

Supplemental Figure S1. PCA plot of study samples and quality control study pool (QCSP) replicates showing clustering and centering of QCSPs, indicating sufficient data quality.
